# Plasma Selenium Biomarkers in Low Income Black and White Americans from the Southeastern United States

**DOI:** 10.1371/journal.pone.0084972

**Published:** 2014-01-20

**Authors:** Margaret K. Hargreaves, Jianguo Liu, Maciej S. Buchowski, Kushal A. Patel, Celia O. Larson, David G. Schlundt, Donna M. Kenerson, Kristina E. Hill, Raymond F. Burk, William J. Blot

**Affiliations:** 1 Department of Internal Medicine, Meharry Medical College, Nashville, Tennessee, United States of America; 2 Division of Gastroenterology, Hepatology, and Nutrition, Department of Medicine, Vanderbilt University School of Medicine, Nashville, Tennessee, United States of America; 3 Department of Public Health, Health Administration and Health Sciences, Tennessee State University, Nashville, Tennessee, United States of America; 4 Department of Population Health, Metro Public Health Department, Nashville, Tennessee, United States of America; 5 Department of Psychology, Vanderbilt University, Nashville, Tennessee, United States of America; 6 Department of Medicine, Vanderbilt University School of Medicine, Nashville, Tennessee, United States of America; University of Texas Health Science Center at San Antonio, United States of America

## Abstract

Biomarkers of selenium are necessary for assessing selenium status in humans, since soil variation hinders estimation of selenium intake from foods. In this study, we measured the concentration of plasma selenium, selenoprotein P (SEPP1), and glutathione peroxidase (GPX3) activity and their interindividual differences in 383 low-income blacks and whites selected from a stratified random sample of adults aged 40–79 years, who were participating in a long-term cohort study in the southeastern United States (US). We assessed the utility of these biomarkers to determine differences in selenium status and their association with demographic, socio-economic, dietary, and other indicators. Dietary selenium intake was assessed using a validated food frequency questionnaire designed for the cohort, matched with region-specific food selenium content, and compared with the US Recommended Dietary Allowances (RDA) set at 55 µg/day. We found that SEPP1, a sensitive biomarker of selenium nutritional status, was significantly lower among blacks than whites (mean 4.4±1.1 vs. 4.7±1.0 mg/L, p = 0.006), with blacks less than half as likely to have highest vs. lowest quartile SEPP1 concentration (Odds Ratio (OR) 0.4, 95% Confidence Interval (CI) 0.2–0.8). The trend in a similar direction was observed for plasma selenium among blacks and whites, (mean 115±15.1 vs. 118±17.7 µg/L, p = 0.08), while GPX3 activity did not differ between blacks and whites (136±33.3 vs. 132±33.5 U/L, p = 0.320). Levels of the three biomarkers were not correlated with estimated dietary selenium intake, except for SEPP1 among 10% of participants with the lowest selenium intake (≤57 µg/day). The findings suggest that SEPP1 may be an effective biomarker of selenium status and disease risk in adults and that low selenium status may disproportionately affect black and white cohort participants.

## Introduction

Over the past eight decades, there has been a great increase in the scientific literature on the health effects of selenium – from its first discovery as a toxic agent in large animals [1], to its recognition as an essential nutrient [2], [3], and to its possible involvement in conditions such as cancer, cardiovascular disease, certain viral infections, immune function, and mood [1], [4]–[6]. Selenium is now known to exist in different tissues and fluids of the body, and in different forms [7], [8], making measurement and interpretation of selenium status difficult.

Selenium has been implicated as a possible inhibitor of a variety of cancers, key among them being prostate [9], colorectal [10], lung [11], esophageal and gastrointestinal cancers [12]. Each of these cancers has a higher incidence and mortality rate in blacks as compared with whites [13], but attempts to correlate these rates with plasma selenium have produced variable results, so, the role of selenium in carcinogenesis, cancer prevention, and cancer disparities has yet to be established [14]–[17]. In the two largest selenium chemoprevention trials, one in China showed a significant reduction in overall cancer mortality among adults supplemented with a combination of selenium (as 50 µg selenium yeast/day), beta carotene and vitamin E [18]. But in the SELECT trial conducted in the US, the hypothesized reduction in the incidence of prostate cancer was not found among those supplemented with 200 µg selenium/day as selenomethionine [19].

Selenium is widely distributed in foods, but its availability depends on the concentration of selenium in the soil. Where there are low selenium soils, selenium deficiency has been demonstrated, especially in China [20], South Africa [21], Poland [22], [23] and other countries [4], [16]. Deficiency is believed not to exist in the US because of the wide distribution of selenium-rich foods. However, the level of selenium in soils in the southeast is lower than in the other parts of the US [24]–[26]. The southeast also comprises a geographic area where the largest numbers of blacks and poor reside [27], and where cancer mortality rates are higher than the rest of the country [28].

It is thought that levels of selenium needed to protect against cancer may be higher than those needed to correct nutrition deficiency [29], [30]. Since the difference between selenium requirement and toxicity is narrow, it is extremely important to define the limits of selenium nutriture [8]. Most frequently measured markers include plasma selenium, SEPP1, and GPX3, which are responsive to changes in selenium intake [8], [20], [31]–[34], and demonstrate functionality of selenium biologically. However, more work is needed in different population groups [8]. Currently published information on the selenium status among low-income residents (both blacks and whites) in the southeastern US is limited.

The purposes of this study were: (1) to measure differences in plasma biomarkers of selenium status (selenium, SEPP1, and GPX3) among low-income blacks and whites selected from a stratified random sample of adults aged 40–79 years, who were participating in a long-term cohort study in the southeastern US, and (2), to determine the utility of plasma biomarkers in assessing selenium status and related demographic, socioeconomic, dietary, and other indicators.

## Materials and Methods

### Ethics Statement

Study protocols were approved by Institutional Review Boards at Meharry Medical College and Vanderbilt University and participants provided written informed consent administered by trained interviewers.

### Populations

#### Southern Community Cohort Study (SCCS)

The SCCS is a prospective cohort study conducted in the southeastern US with a major goal to understand the causes of cancer and other key health disparities among blacks and whites. Details of the methods have been described elsewhere [35, [36]. In brief, adults were recruited from community health centers (CHCs) across a 12-state region (Alabama, Arkansas, Florida, Georgia, Kentucky, Louisiana, Mississippi, North Carolina, South Carolina, Tennessee, Virginia, and West Virginia). Since CHCs provide basic and preventive health care in underserved areas, most of the black and white enrollees were of low socio-economic status [37]. Eligible participants were 40 to 79 years of age, had not been diagnosed with or under treatment for cancer for at least the past year, and were English speaking. After consenting, participants were administered a comprehensive computer-assisted interview by a trained interviewer asking for demographic, anthropometric, medical history, diet, physical activity, use of tobacco, alcohol, medications, and other characteristics. Biologic specimens (blood, saliva, and/or urine samples) were provided by approximately 90% of the cohort participants recruited from the CHCs. Blood was provided by 54%, with generally similar characteristics of those with vs. those without blood donation [36].

To help characterize the cohort, a series of biomarker studies were carried out using biospecimens from a stratified random sample of SCCS participants [38]–[41]. A 2×2×3×3 factorial design was used to draw the sample and ensure even balance by race (black/white), sex (male/female), smoking (never/former/current), and body mass index (BMI) (<25.0, 25.0–29.9, ≥30 kg/m^2^) so that differences in biomarkers between these groups could be detected with substantially enhanced statistical power. For the selenium biomarker studies reported herein, 383 participants were included (Table 1).

**Table 1:** Characteristics of the SCCS sample.

Demographics Variables		Whites	Blacks
**Gender**			
	Male	96 (50)	94 (49)
	Female	95 (50)	98 (51)
**Age Group (years)**			
	40 to 49	67 (35)	101 (53)
	50 to 59	65 (34)	57 (30)
	60 to 79	59 (31)	34 (18)
**Education**			
	< High School	55 (29)	69 (36)
	High School	66 (35)	67 (35)
	>High School	70 (37)	56 (29)
**Household Annual Income**			
	Less than $15,000	116 (61)	113 (59)
	$15,000 – <$25,000	40 (21)	44 (23)
	$25,000 – <$50,000	21 (11)	31 (16)
	$50,000 or more	13 (7)	2 (1)
	Missing	1 (1)	2 (1)
**BMI (kg/m^2^)**			
	18.3 – 24.9	63 (33)	63 (33)
	25.0 – 29.9	65 (34)	65 (34)
	≥30.0	63 (33)	64 (33)
**Lifestyle Variables**		**Whites**	**Blacks**
**Cigarette smoking**			
	Non Smoking	63 (33)	64 (33)
	Former Smoking	65 (34)	65 (34)
	Current Smoking	63 (33)	63 (33)
**Fruit and vegetable intake (times/day)**			
	0 – 1	30 (16)	22 (11)
	2 – 4	99 (52)	79 (41)
	At least 5	62 (32)	91 (47)
**Meat and fish intake (times/day)**			
	0 – 1	111 (58)	83 (43)
	2 – 4	75 (39)	87 (45)
	At least 5	5 (3)	22 (11)
**Living on farm**			
	Yes	108 (57)	79 (41)
	No	83 (43)	113 (59)
**Outcome Variables**		**Whites**	**Blacks**
**Se**		188 (98)	190 (99)
	Missing	3 (2)	2 (1)
**SEPP1**		191 (100)	192 (100)
	Missing		
**GPX3**		184 (96)	182 (95)
	Missing	7 (4)	10 (5)

BMI - Body Mass Index; SEPP1 - Selenoprotein P; GPX3 - Glutathione Peroxidase.

#### The Third National Health and Nutrition Examination Survey (NHANES III)

To investigate how SCCS selenium results compared with other population data, and to compare newly assessed selenium status for the general population of the southern region, we accessed data from NHANES III (1988–1994) (NHANES III, series 11, no. 1A, http://www.cdc.gov/nchs/nhanes/nh3data.htm#1a). NHANES III is a US nationwide survey conducted by the National Center for Health Statistics of the Centers for Disease Control and Prevention, designed to provide national estimates of the health and nutritional status of the United States' civilian, non-institutionalized population. For comparison with the SCCS data, we obtained data for 2,567 participants in the NHANES III database with characteristics similar to the SCCS population: 40 to 79 years of age, living in the southeastern region of the US, being non-Hispanic white or non-Hispanic black, having a plasma selenium measure, and not being pregnant. We compared only plasma selenium concentration since NHANES III does not report SEPP1 or GPX3 data.

### Selenium Biomarker Measures

Blood samples for SCCS participants were collected in two 10-ml tubes, one with EDTA preservative, from non-fasting study subjects at baseline at the CHCs. The vast majority (>95%) of blood samples were processed at Vanderbilt University within 24 hours of collection, separated into aliquots of serum, plasma, red blood cells, buffy coat, and clot, using standard techniques, and kept frozen at −80°C. A plasma aliquot was used for the selenium biomarker assays.

Plasma selenium status was assessed using the three biomarkers of selenium status in our laboratory at Vanderbilt University, Nashville:

(i) Selenium was measured fluorometrically, using methods of Koh and Benson [42], as modified by Sheehan and Gao [43], and expressed as µg/L (µmol/L).

(ii) SEPP 1 was measured by ELISA and expressed as mg/L. A standard curve using purified human selenoprotein P was used for calibration daily [44], and

(iii) GPX3 activity was expressed as U/L. One unit equals one µmol NADPH oxidized per minute [32]. The method measures the rate of oxidation of NADPH by glutathione peroxidase, using 0.25 mM H_2_O_2_ as substrate in the presence of 2 mM GSH.

Within NHANES III, plasma selenium was measured by atomic absorption spectrophotometry. It has been shown before that there is no difference in measurement outcomes between atomic absorption spectrophotometry and the fluorometric method used by Burk and Hill [31], [43], [45].

### Dietary Selenium Intake

Daily dietary selenium intakes (µg/day) in SCCS participants were estimated using the 89-item Food Frequency Questionnaire (FFQ), developed empirically to include an optimal number and selection of foods that would capture the main sources of energy and key nutrient intakes in the SCCS population [46]. Selenium content in food items eaten was estimated based on sex-, race-, and census region-specific food lists using 24-hour recall data from NHANES III, NHANES 1999–2000, NHANES 2001–2002, NHANES 2003–2004, and the Continuing Survey of Food Intakes by Individuals (CSFII 1994–96). This approach enabled estimation of selenium intake tailored to the participant characteristics and to the southern diets typically eaten by potential SCCS black and white participants [47]. An estimate of selenium intake was available for all SCCS participants with measured plasma selenium biomarkers, but dietary selenium estimates were not available for NHANES III participants.

### Statistical Analyses

Among 383 study participants, 377 had selenium, 383 had SEPP1, and 366 had GPX3 plasma concentrations measured, and all participants were characterized by demographic and lifestyle variables according to race groups (Table 1). The *t* test and analysis of variance (ANOVA) models were used to test the equality in the mean values for the selenium biomarkers across demographic and lifestyle variables (Table 2). Tukey's *post hoc* analysis for multiple comparisons was used to determine the significant mean differences for more than two levels of the demographic and lifestyle variables. Correlations between the selenium biomarkers were analyzed using the Pearson correlation coefficient (Table 3).

**Table 2:** Mean difference by sociodemographic and lifestyle variables for selenium biomarkers among SCCS participants.

Demographics		Selenium (µg/L)				SEPP1 (mg/L)				GPX3 (U/L)			
		N	Mean	(SD)	p value	N	Mean	(SD)	p value	N	Mean	(SD)	p value
**Race**													
	White	188	117.6	(17.7)	0.082	191	4.7	(1.0)	0.006	184	132.4	33.5	0.320
	Black	189	114.6	(15.1)		192	4.4	(1.1)		182	135.8	33.3	
**Sex**													
	Male	187	116.5	(15.1)	0.591	190	4.7	(1.1)	0.010	182	134.9	35.0	0.636
	Female	190	115.6	(17.8)		193	4.4	(1.1)		184	133.3	31.8	
**Age group (years)**													
	40 to 49	165	113.8	(14.8)	0.004	168	4.3	(1.0)	0.001	160	136.9	34.5	0.315
	50 to 59	120	120.1	(18.6)		122	4.8	(1.2)		116	133.0	33.8	
	60 to 79	92	114.9	(15.7)		93	4.7	(1.0)		90	130.5	30.7	
**Education**													
	< High School	119	116.5	(15.6)	0.811	124	4.7	(1.3)	0.079	119	135.3	35.7	0.880
	High School	132	115.3	(17.6)		133	4.4	(1.0)		124	133.8	31.2	
	> High School	126	116.5	(16.2)		126	4.5	(0.9)		123	133.2	33.4	
**Household Income**													
	Less than $15,000	224	115.8	(17.4)	0.972	229	4.5	(1.1)	0.408	219	133.8	33.7	0.935
	$15,000 – <$25,000	83	116.2	(15.7)		84	4.6	(1.1)		80	135.6	31.8	
	$25,000 – <$50,000	52	116.5	(15.4)		52	4.4	(1.0)		50	133.8	35.5	
	$50,000 or more	15	117.8	(11.8)		15	4.8	(0.9)		14	129.8	28.6	
**BMI (kg/m^2^)**													
	18.3 – 24.9	123	116.7	(20.1)	0.775	126	4.5	(1.3)	0.188	122	137.8	35.0	0.171
	25.0 – 29.9	128	115.3	(14.5)		130	4.5	(1.1)		122	134.6	32.4	
	≥30.0	126	116.2	(14.5)		127	4.7	(0.9)		122	129.8	32.5	
**Cigarette smoking**													
	Non smoking	125	119.5	(18.7)	<0.001	127	4.6	(1.2)	0.250	122	136.9	33.0	0.215
	Former smoking	128	118.4	(16.2)		130	4.6	(1.0)		125	135.5	34.0	
	Current smoking	124	110.2	(12.5)		126	4.4	(1.0)		119	129.8	33.0	
**Fruit and vegetable intake (servings/day)**													
	0 – 1	50	113.4	(14.9)	0.441	52	4.5	(0.9)	0.595	49	131.1	33.2	0.744
	2 – 4	177	116.8	(16.1)		178	4.6	(1.2)		172	135.2	34.5	
	At least 5	150	116.1	(17.4)		153	4.5	(1.0)		145	133.8	32.2	
**Meat and fish intake (servings/day)**													
	0 – 1	191	116.1	(16.7)	0.793	194	4.5	(1.0)	0.317	186	136.1	35.2	0.437
	2 – 4	159	116.4	(16.4)		162	4.6	(1.2)		153	132.4	31.4	
	At least 5	27	114.1	(15.9)		27	4.4	(1.2)		27	129.2	31.7	
**Living on farm**													
	Yes	183	117.2	(15.5)	0.186	187	4.6	(0.9)	0.448	177	135.2	33.0	0.520
	No	194	115.0	(17.4)		196	4.5	(1.2)		189	133.0	33.8	

BMI - Body Mass Index; SEPP1 - Selenoprotein P; GPX3 - Glutathione Peroxidase.

**Table 3:** Relationships between plasma Selenium, (SEPP1), and GPX3) in the SCCS sample.

	*r*	p value
**Se - SEPP1**	0.49	<0.001
**Se - GPX3**	0.17	0.001
**SEPP1 - GPX3**	0.05	0.387

SEPP1 - Selenoprotein P; GPX3 - Glutathione Peroxidase.

The 25th, 50th, and 75th selenium biomarker percentiles were used as cut-off points to establish quartile distributions (Table 4). Multivariate logistic regression models for the biomarkers were then utilized to calculate the odds ratios and 95% CIs for blacks vs. whites at the higher quartiles (Q2, Q3, and Q4), using the lowest quartile (Q1) as the reference. All other demographic (sex, age, education level, household annual income, and BMI) and lifestyle (smoking status, fruit and vegetable intake, meat and fish intake, and living on farm) variables were adjusted in these models. The Mantel-Haenszel trend test was used to test the linear trend in odds ratios across the quartiles by race.

**Table 4:** Plasma selenium concentration odds ratios for black and white Americans participating in the SCCS.

Quartile of plasma Selenium concentration											
	Q1 (low)†		Q2		Q3		Q4 (high)			mean (SD)	median (range)
	n	Odds ratio‡	n	Odds ratio	n	Odds ratio	n	Odds ratio	p value		
**Selenium (µg/L)**											
Range	(77.0–105.3)		(105.4–113.9)		(114.0–123.7)		(123.8–192.6)			116.1 (16.5)	114.0 (77.0–192.6)
White	44	1.0	41	1.0	50	1.0	53	1.0		117.6 (17.7)	115.8 (77.0–192.6)
Black	50	1.0	54	1.1 (0.6 2.0)	43	0.9 (0.5 1.6)	42	0.7 (0.4 1.4)	0.103	114.6 (15.1)	113.1 (82.9–168.0)
**Selenoprotein P (mg/L) (SEPP1)**											
Range	(1.1–3.9)		(3.9–4.5)		(4.5–5.1)		(5.1–12.4)			4.6 (1.1)	4.5 (1.1–12.4)
White	33	1.0	49	1.0	53	1.0	56	1.0		4.7 (1.0)	4.6 (2.2–9.4)
Black	62	1.0	45	**0.5 (0.3 0.9)**	45	**0.4 (0.2 0.8)**	40	**0.4 (0.2 0.8)**	0.002	4.4 (1.1)	4.3 (1.1–12.4)
**Glutathione Peroxidase (GPX3) (U/L)**											
Range	(59.5–110.6)		(110.6–131.6)		(131.7–155.8)		(155.8–264.8)			134.1 (33.4)	131.7 (59.5–264.8)
White	48	1.0	54	1.0	41	1.0	41	1.0		132.4 (33.5)	125.4 (59.5–264.8)
Black	43	1.0	38	0.8 (0.4 1.5)	50	1.6 (0.9 3.1)	51	1.4 (0.7 2.6)	0.102	135.8 (33.3)	136.1 (62.3–242.9)

SEPP1 - Selenoprotein P; GPX3 - Glutathione Peroxidase

†Quartile cutpoints were based on the distribution for each selenium (Se) plasma concentration with whites and blacks combined.

‡All models were adjusted for gender, age, education, household income, body mass index, smoking status, fruit and vegetable intake, meat and fish intake, and living on farm.

The mean value and standard deviation of SEPP1 and GPX3 within each quartile of selenium were calculated (Table 5), and significant differences were determined by the *t* test.

**Table 5:** Mean SEPP1 and GPX3 plasma concentration according to quartiles of selenium concentration stratified by race.

Quartiles of the plasma selenium concentration						
Biomarker	Race		Q1 (low)†	Q2	Q3	Q4 (high)
**Selenium (µg/L)**						
		**Range**	**(77.0–105.3)**	**(105.4–113.9)**	**(114.0–123.7)**	**(123.8–192.6)**
		**Mean.(SD)**	97.6±6.2	110.3±2.6	118.9±2.8	137.5±13.8
**SEPP1 (mg/L)**			**Mean (SD)**	**Mean (SD)**	**Mean (SD)**	**Mean (SD)**
	**Whites**	**Mean (SD)**	4.1±0.8	4.5±0.9	4.8±0.8	5.2±1.1
		**n**	44	41	50	53
	**Blacks**	**Mean (SD)**	3.9±0.8	4.3±1.1	4.4±0.8	5.0±1.0
		**n**	50	54	43	42
**GPX3 (U/L)**			**Mean (SD)**	**Mean (SD)**	**Mean (SD)**	**Mean (SD)**
	**Whites**	**Mean (SD)**	126.8±31.1	126.1±25.7	132.5±32.8	139.6±39.4
		**n**	43	39	48	51
	**Blacks**	**Mean (SD)**	122.2±26.9	142.2±36.1	131.7±31.1	147.9±33.3
		**n**	48	52	42	39

SEPP1 - Selenoprotein P; GPX3 - Glutathione Peroxidase.

†Quartile cutpoints were based on the distribution for the Selenium concentration with whites and blacks combined.

The plasma selenium of the SCCS participants was compared with plasma selenium in the NHANES III sample (Table 6). Selenium concentration means were calculated within each group categorized by race, sex, age, education level, household annual income, and BMI, by using the Surveymeans program in SAS after weighting of the NHANES III sample. The *t* test was used to compare the mean difference between the SCCS and the NHANES III participants.

**Table 6:** Comparison of plasma selenium concentration between the SCCS and NHANES III (1988–1994) participants.

		Selenium (µg/L)				*t*	p-value
		SCCS^*^		NHANES III			
		N	Mean (SD)	N	Mean (SD)		
**Race**							
	Whites	188	117.6 (17.7)	1472	121.5 (33.7)	1.6	0.118
	Blacks	189	114.6 (15.1)	1095	117.4 (32.4)	1.2	0.238
**Sex**							
	Male	187	116.5 (15.1)	1258	122.4 (32.6)	2.4	0.015
	Female	190	115.6 (17.8)	1309	119.4 (29.4)	1.7	0.081
**Age group (years)**							
	40–49	165	113.8 (14.8)	771	121.7 (19.7)	4.9	<0.001
	50–59	120	120.1 (18.6)	604	121.6 (26.0)	0.6	0.555
	60–79	92	114.9 (15.7)	1192	119.3 (45.6)	0.9	0.357
**Education**							
	<High School	119	116.5 (15.6)	1134	118.7 (38.5)	0.6	0.528
	High School	132	115.3 (17.6)	737	120.1 (30.5)	1.8	0.081
	>High School	126	116.5 (16.2)	647	123.7 (15.2)	4.8	<0.001
**Household Income**							
	Less than $15,000	224	115.8 (17.4)	785	117.1 (50.7)	0.4	0.706
	$15,000–<$25,000	83	116.2 (15.7)	523	118.6 (17.6)	1.2	0.228
	$25,000–<$50,000	52	116.5 (15.4)	649	122.2 (30.1)	1.4	0.177
	$50,000+	15	117.8 (11.8)	371	124.1 (18.1)	1.3	0.187
**BMI (kg/m^2^)**							
	18.3–24.9	123	116.7 (20.1)	846	122.5 (26.7)	2.3	0.021
	25.0–29.9	128	115.3 (14.5)	954	120.3 (33.2)	1.7	0.090
	≥30.0	126	116.2 (14.5)	732	119.3 (25.1)	1.3	0.183
						
	All	377	116.1 (16.5)	2567	120.9 (40.5)	2.3	0.023

NHANES III: 40–79 yrs, South region of US.; BMI - Body Mass Index.

To compare the selenium biomarkers with the dietary selenium intakes in 351 participants who had both biomarker and dietary data, the Pearson correlation coefficient (*r*) was used (Table 7). After excluding 3 participants with missing covariate information, an adjusted Pearson correlation coefficient (adjusted *r*) was computed controlling for potential confounding by age (years, continuous), BMI (kg/m2, continuous), race (black and white), sex (male and female), household annual income (≤$15,000; $15,001-<$25,000; $25,001-<$50,000; and >$50,001), education level (<high school, high school, >high school), (smoking status (non-smoker, former smoker, current smoker), and living on a farm or in an urban area (discrete). The sample was divided into two groups below or above the 10^th^ percentile of the daily selenium intake (≤57 µg/day) using the US RDA set at 55 µg/day as a criterion [48]. The Pearson correlation coefficients (*r*, adjusted *r*) were applied to test the linear relationship between the plasma selenium concentrations with estimated daily selenium intakes for the two selenium intake groups.

**Table 7:** Correlation and partial Pearson correlation coefficients testing the strength of the linear association between daily selenium intake and three plasma selenium biomarkers in SCCS participants.

	Selenium	SEPP1	GPX3
**Total sample (N = 351)**			
**Crude ** ***r***	0.01	−0.01	−0.06
**Adjusted ** ***r*** **†**	0.00	−0.02	−0.12*
**Daily selenium intake ≤57 (µg/day)(N = 36) group**			
**Crude ** ***r***	0.31	0.41*	−0.04
**Adjusted ** ***r*** **†**	0.36	0.56**	0.05
**Daily selenium intake >57 (µg/day)(N = 315) group**			
**Crude ** ***r***	0.00	−0.03	−0.04
**Adjusted ** ***r*** **†**	−0.01	−0.03	−0.11

Adjusted r†: adjusted for age, BMI (continuous), race, gender, education, household annual income, smoking status, and living on a farm (discrete).

* : p<0.05; **: p<0.005.

BMI - Body Mass Index; SEPP1 - Selenoprotein P; GPX3 - Glutathione Peroxidase.

Statistical analyses were performed using SAS version 9.2 (SAS Institute, Cary, NC). All statistical tests were based on a two-sided probability, and p<0.05 was considered significant.

## Results

### Characteristics of the SCCS Study Population

By design, the present sample was balanced by race (black, n = 191 and white, n = 192), sex (male and female; 50% each), obesity status (BMI-18.3-24.9, 25.0–29.9, 30.0–44.9 kg/m2; 33% in each category), and smoking status (non-smoker, former smoker, current smoker; 33% in each category). This balance was present in these variables when categorized by race (Table 1). Additionally, education level (<high school, high school, >high school) was roughly divided by one-third in each category. Other variables not distributed evenly by race included: age, with 35% white and 53% black between 40 and 49, 34% white and 30% black between 50 and 59, and 31% white and 18% black between 60 and 79 years of age. The socio-economic status of the sample was low, with 61% white and 59% black making less than $15,000; 21% white and 23% black, $15,000-<$25,000; 11% white and 16% black, $25,000-<$50,000; and 7% white and 1% black, $50,000 or more. Among dietary factors, we considered fruit and vegetable intake, with 16% white and 11% black, 52% white and 41% black, and 32% white and 47% black reporting an intake of 0–1, 2–4, and at least 5 servings per day, respectively; and meat and fish intake, with 58% white and 43% black, 39% white and 45% black, and 3% white and 11% black reporting an intake of 0–1, 2–4, and at least 5 servings per day, respectively. Fifty-seven percent of whites and forty-one percent of blacks had lived in a rural community or farm, and 43% whites and 59% blacks had lived in an urban area.

### Effect of SCCS Socio-Demographic and Lifestyle Factors on Selenium Biomarkers

#### Selenium (µg/L)

There were significant differences in plasma selenium concentration across age groups (p = 0.004) and smoking status groups (p<0.001) (Table 2). The plasma selenium in the 40–49 year group was significantly lower than that in the 50–59 year group (p = 0.0016), but there was no monotonic trend with age. Current smokers had plasma selenium significantly lower than those who never smoked (p = 0.0001) and former smokers (p = 0.0001). Blacks had a slightly lower plasma selenium than whites (114.6 vs117.6, p = 0.08). No significant differences were found by education, income, BMI, rural vs. urban residence, fruit and vegetables, nor meat and fish consumption.

#### Selenoprotein P (SEPP1, mg/L)

Significant differences in plasma SEPP1 were found in race (p = 0.006), sex (p = 0.01), and age groups (p = 0.001), with lower SEPP1 means among blacks than whites, females than males, and those of age group 40–49 years, but with no monotonic trend with age (Table 2). The mean level in blacks was 0.3 mg/L lower than in whites. There were no significant differences by education, income, BMI, rural vs. urban residence, fruit and vegetables, nor meat and fish consumption.

#### Glutathione Peroxidase (GPX3 Activity) (U/L)

No significant associations were found between mean GPX3 and tested demographic and lifestyle variables (Table 2). Also, the mean GPX3 activity was not different between blacks and whites (135.8 U/L vs. 132.4 U/L, p = 0.320).

### Correlations of the plasma selenium biomarkers

Selenium plasma concentrations were significantly correlated with SEPP1 (*r* = 0.49, p<0.001) and GPX3 (*r* = 0.17, p = 0.001), but there was no correlation between SEPP1 and GPX3 concentration (*r* = 0.05, p = 0.39) (Table 3).

### Black vs. White Differences in Selenium Biomarker Status by Quartile Distribution

#### Selenium

Plasma selenium concentration ranged from 77.0 µg/L (0.98 µmol/L) to 192.6 µg/L (2.45 µmol/L) (mean ± SD, 116.1±16.5 µg/L (1.47±0.21 µmol/L); median, 114.0 µg/L (1.45 µmol/L)) (Table 4). The 1^st^ quartile was from 77.0 to <105.3 µg/L, the 2^nd^ quartile from ≥105.4 to <113.98 µg/L, the 3^rd^ quartile from ≥114.0 to <123.7 µg/L, and the 4^th^ quartile from ≥123.8 to 192.6 µg/L. The odds of having high plasma selenium tended to be lower among blacks than whites (4^th^ quartile), but the difference was not significant (p for trend  = 0.103).

#### Selenoprotein P (SEPP1)

Plasma SEPP1 concentration ranged from 1.1 mg/L to 12.4 mg/L (mean±SD, 4.6±1.1 mg/L; median, 4.5 mg/L) (Table 4). The odds of having high SEPP1 level were significantly lower for blacks than whites (p for trend  = 0.002).

#### Glutathione Peroxidase Activity (GPX3)

Plasma GPX3 concentration ranged from 59.5 U/L to 264.8 U/L (mean±SD, 134.1±33.4 U/L; median, 131.7 U/L) (Table 4). Blacks tended to have higher GPX3 activity than whites, but the differences were not significant (p for trend  = 0.102).

### Comparison of SEPP1 and GPX3 across Plasma Selenium Quartiles

There was a significant difference in mean SEPP1 levels (p = 0.0004) across plasma selenium quartiles 3 and 4, with SEPP1 levels rising monotonically with selenium concentration among both blacks and whites (Table 5). There was a significant difference between GPX3 levels across selenium quartiles 1 and 2 among blacks (p = 0.0022), but not significantly different thereafter across the remaining quartiles for blacks or whites.

### Comparison of SCCS and NHANES III Mean Selenium Levels

The average plasma selenium concentration in SCCS was significantly lower than that reported by NHANES III for the southeastern US (p = 0.023) (Table 6). The lower concentrations held among both blacks and whites and men and women and in most remaining demographic strata, with the largest SCCS vs. NHANES III differences (≥6 µg/L (0.08 µmol/L) among males (p = 0.015), in those aged 40–49 years of age (p<0.001), those with greater than a high school education (p<0.001), and those with normal BMI (p = 0.021). However, there were no significant differences between the other SCCS and NHANES III variables. Within NHANES III, mean selenium concentration was lower among blacks than whites (p = 0.002) and females than males (p = 0.001), and showed stronger gradients of increasing levels with rising education and income than in the SCCS. Within the SCCS, significant differences by race and gender were reflected in the SEPP1, and not the selenium, measures (Table 2).

### Correlations between Daily Intakes of Selenium with Selenium Biomarkers

No overall linear relationships between the three selenium biomarkers and estimated daily selenium intake were found (Table 7). When participants were divided into two subgroups based upon whether their estimated daily dietary selenium intake was ≤57 µg/day or >57 µg/day (10^th^ percentile), a significant linear relationship was found between SEPP1 and daily selenium intake among participants in the ≤57 µg/day group (*r* = 0.41, p<0.05; adjusted *r* = 0.56, P<0.005). None of the selenium biomarkers was significantly related to higher levels (>57 µg/day) of estimated daily selenium intake. The significance displayed for GPX3 (adjusted *r* = −0.12, p<0.05) in the total sample was not repeated in the two subgroups.

## Discussion

In this new examination of three biomarkers of selenium status (plasma selenium, SEPP1 and GPX3) in a low-income southeastern US population, we found that SEPP1 provides the most discrimination in identifying those with high vs. low selenium status and in detecting differences between blacks and whites. We compared plasma selenium results in our study with the NHANES III southern regional population sample and found that plasma selenium levels among the SCCS participants were slightly or significantly lower than the NHANES III population within the strata of some demographic variables (Table 6).

Since the NHANES III did not measure SEPP1, we compared its concentration in the SCCS population with the baseline values from the 81 healthy adults (mean age 36 years, 73% female) entering a selenium supplementation trial at Vanderbilt University [31]. The overall mean values in the SCCS were lower for selenium (116 vs.121 µg/L, (1.47 vs. 1.54 µmol/L) p<0.002)), SEPP1 (4.5 vs. 5.3 mg/L, p<0.0001), and GPX3 activity (134 vs. 159 U/L, p<0.0001). It is possible that these differences were associated with the lower SES and race differences between the SCCS and Vanderbilt trial participants.

Prior research assessing SEPP1 has been limited. Burk and colleagues reported an SEPP1 concentration of 5.3±0.9 mg/L among healthy adults participating in the Vanderbilt trial [20], [31], and Saito et al. [49] reported 5.3±1.1 mg/L plasma SEPP1 in 73 healthy Japanese. Hurst et al [50] reported that in a 12-week supplementation trial, mean SEPP1 in 119 healthy British men and women aged 50–64 years of age increased from a 4.99±0.80 µg/ml at baseline to 6.17±0.85, 6.73±1.01 and 6.59±0.64 µg/ml depending on the supplemental selenomethionine dose (50, 100, and 200 µg, respectively). These figures are within the range of normal by the Burk and Xia studies. Xia et al. [33] also reported that plasma SEPP1 was maximized in deficient Chinese at similar levels (from 4.9±1.1 to 5.6±1.1 mg/L) (n = 14/group) when supplemented with 35, 55, 79, 102, and 125 µg/day selenomethionine, over 4 to 40 weeks. The time of optimization varied with the dose and length of supplementation. In our study with the largest population sample reported to date in the US (n = 383), SEPP1 ranged from 1.0 to 12.4 mg/L, with a mean level of 4.6±1.1 mg/L, and half the participants having SEPP1 at or below 4.5 mg/L.

SEPP1 and GPX3 are the only two known plasma selenoproteins, with SEPP1 representing over 50% of plasma selenium, and GPX3 10–30% [51]; more recent work by Combs et al [52] indicate that these values may be closer to 34% and 20%, respectively. SEPP1, an extracellular protein produced in the liver is believed to be important in performing specific transport functions from the liver to peripheral tissues and in protecting against oxidative injury [33], [49], [51], [53]. GPX3 is a plasma protein synthesized in the proximal tubular cells of the kidney, reducing hydroperoxides to harmless products (water and alcohol) [33], [51]. Both plasma SEPP1 and GPX3 are depressed in selenium deficiency and reach a plateau when they achieve their optimal functional activity [53]. The levels of maximization/optimization vary by biomarker, amount of selenium available, and time required to perform their specific functions [33].

The SEPP1 in plasma has been postulated to be a more appropriate measure of selenium nutritional status than either plasma selenium or plasma GPX3 due to its need for higher levels of selenium for optimization [54]. Because of its transport function, SEPP1 is believed to “fill up body selenium pools sequentially” [33], so would be a more sensitive overall indicator than GPX3, which is synthesized in the kidney [33], [54]. There is considerable uncertainty, however, regarding the selenium concentration needed to achieve maximization of SEPP1 or GPX3 (i.e. concentrations associated with adequate selenium nutritional status), and whether these vary across black or white populations. Selenium levels for GPX3 maximization have been reported to vary from 40–200 µg/L (0.51–2.54 µmol/L) [55]; 70–100 µg/L (0.89–1.27 µmol/L) [56]; 80–90 µg/L (1.02–1.14 µmol/L) [33]; 91–122 µg/L (1.16–1.55 µmol/L) [57]; and 89–114 µg/L (1.13–1.45 µmol/L) [16], but comparable data for SEPP1 are sparse. While our results are not robust enough to demonstrate cut-off points for maximization of either GPX3 or SEPP1, we found that SEPP1 levels rose monotonically across ascending plasma selenium quartiles in blacks and whites, meaning possibly that SEPP1 was increasing towards, but not achieving, optimization in these subjects (Table 5). On the other hand, the GPX3 levels in blacks rose significantly from selenium quartile 1 to 2, then dropped and rose again (non-significantly) in quartile 4, while in whites, they rose non-significantly to quartile 4. These patterns might be perhaps suggestive of maximization of SEPP1 and GPX3 at different time points at selenium concentrations above 100 µg/L (the upper bound level of selenium quartile 1), with possible differences by race.

Using plasma selenium cutoffs of 80, 90, 105, and 114 µg/L as potential levels for GPX3/SEPP1 maximization, the proportions of the SCCS sample that may be considered to have insufficient selenium status for functional activity would be 1%, 5%, 25% and 50% respectively. Blacks tended to have higher insufficiency percentages than whites at 90, 105, and 114 µg/L (6vs.3%, 28vs.24%, and 55vs.45% respectively). Furthermore, at selenium levels above 105 µg/L (1.33 µmol/L), the mean SEPP1 concentrations for both blacks and whites in the SCCS were below 5.3 mg/L (the US normal level) (Table 4) [54], indicating that SEPP1 had not been maximized, and raising the possibility that a marginal selenium insufficiency, by the SEPP1 measure, may not be uncommon in these populations.

Among the biomarkers, SEPP1 showed the strongest racial differences, with blacks having mean SEPP1 significantly lower than whites (p = 0.006) (Table 2). We also found that SEPP1 levels varied significantly by sex and age groups. Plasma selenium was marginally lower among blacks than whites and significantly lower among smokers than non-smokers, and younger (39–49 years old) age groups. GPX3 activity was not associated with race, gender, age, or smoking status. Prior NHANES III data analyses reported lower plasma selenium levels among blacks than whites, females than males, and smokers than non-smokers [24], [58], [59]. We confirmed that these patterns held within a subgroup of NHANES III participants living in the southeastern US. In our study, plasma selenium levels were significantly associated with both SEPP1 and GPX3, while SEPP1 was not correlated with GPX3, suggesting separate functions for these selenoproteins.

Although none of the biomarkers showed an overall association with estimated daily dietary selenium intake, we found that SEPP1 was positively correlated with selenium intake in the subset of SCCS participants with intake ≤57 µg/day (10^th^ percentile crude *r* = 0.41, p<0.05; adjusted *r* = 0.56, p<0.005) (Table 7). Our findings showing SEPP1 association with race, sex, and lower dietary selenium intake suggest that SEPP1 may be the most sensitive biomarker of selenium status in our study population. These findings are consistent with previous reports by Burk and colleagues (20, 31, 33) who concluded that SEPP1 is the most sensitive predictor of selenium nutritional status.

### Implications and Conclusions

The primary purpose of this study was to determine whether there may be variation in selenium status and to provide clues to differentials in indications of selenium insufficiency among black and white participants in the SCCS. This study demonstrated the feasibility of assessing selenium status using three plasma selenium biomarkers in a representative sample of low income black and white adults. SEPP1 values in this group were slightly below those in more affluent strata [30], suggesting that there may be marginal selenium deficiency in more than half of the SCCS population. The association of selenium nutritional status with plasma SEPP1 was higher than with selenium and GPX3. This suggests that future studies, including planned assessments of an association of baseline selenium status with cancer incidence among SCCS participants focus on this biomarker.
